# Dietary fat induced chylomicron-mediated LPS translocation in a bicameral Caco-2cell model

**DOI:** 10.1186/s12944-022-01754-3

**Published:** 2023-01-12

**Authors:** Monic M. M. Tomassen, Coen Govers, A. Paul Vos, Nicole J. W. de Wit

**Affiliations:** 1 Wageningen Food & Biobased Research, Wageningen University & Research, Wageningen, The Netherlands; 2 Wageningen Food & Biobased Research – Food Health & Consumer Research group, Bornse Weilanden 9, 6708 WG Wageningen, The Netherlands; 3 Cell Biology and Immunology Group, Wageningen University & Research, Wageningen, The Netherlands

**Keywords:** Lipopolysaccharide, Dietary fat, Chylomicrons, Intestine, Translocation

## Abstract

**Background:**

There is increasing evidence that dietary fat, especially saturated fat, promotes the translocation of lipopolysaccharide (LPS) via chylomicron production in the gut. Chylomicrons can subsequently transport LPS to other parts of the body, where they can induce low-grade chronic inflammation that is linked to various metabolic and gut-related diseases. To identify promising (food) compounds that can prevent or ameliorate LPS-related low-grade inflammation, we developed and optimized a bicameral in vitro model for dietary fat-induced LPS translocation that closely mimics the in vivo situation and facilitates high-throughput screening.

**Methods:**

Caco-2 cells were cultured in monolayers and differentiated to a small intestinal phenotype in 21 days. Thereafter, optimal conditions for fat-induced chylomicron production were determined by apical exposure of Caco-2 cells to a dilution range of in vitro digested palm oil and sunflower oil, optionally preceded by a 1-week apical FBS deprivation (cultured without apical fetal bovine serum). Chylomicron production was assessed by measuring basolateral levels of the chylomicron-related marker apolipoprotein B. Next, LPS was coincubated at various concentrations with the digested oils, and fat-induced LPS translocation to the basolateral side was assessed.

**Results:**

We found that dietary fat-induced LPS translocation in Caco-2 cells was optimal after apical exposure to digested oils at a 1:50 dilution in combination with 750 ng/mL LPS, preceded by 1 week of apical FBS deprivation. Coincubation with the chylomicron blocker Pluronic L81 confirmed that fat-induced LPS translocation is mediated via chylomicron production in this Caco-2 cell model.

**Conclusion:**

We developed a robust Caco-2 cell model for dietary fat-induced LPS translocation that can be used for high-throughput screening of (food) compounds that can reduce LPS-related low-grade inflammation.

**Supplementary Information:**

The online version contains supplementary material available at 10.1186/s12944-022-01754-3.

## Background

There is growing evidence for a role of the intestinal barrier in systemic low-grade inflammation [1, 2]. Impaired intestinal barrier function facilitates the passage of luminal antigens or other molecules, which in turn can provoke a local or systemic immune response. A luminal antigen that is commonly linked to chronic inflammation is lipopolysaccharide (LPS), which consists of a lipid A moiety, an inner- and outer core and a long-chain O-antigen polysaccharide. LPS is a cell wall component of gram-negative bacteria that is naturally present in the gut in high quantities [3, 4]. LPS can cross the intestinal barrier from the intestinal lumen via paracellular and/or transcellular routes. Paracellular translocation can occur when tight junction functioning is impeded, resulting in reduced intestinal integrity. For example, ethanol induces disruption of tight junctions and can therefore enforce paracellular translocation of LPS [5, 6], which can subsequently contribute to alcoholic liver disease (ALD) [7]. Transcellular translocation of LPS, on the other hand, is especially linked to a high load of dietary fat [8]. In vitro, animal and human studies have demonstrated that the high intake of dietary fat, especially saturated fat, facilitates the translocation of luminal LPS across the intestinal barrier. Chylomicrons, functioning as transporter molecules of dietary triglycerides, seem to play an important role in this process, as after a high-fat challenge, postprandial LPS levels in blood peak at the same time as chylomicron levels, and LPS shows the highest concentration in the chylomicron-rich fraction [9–14]. Via chylomicrons, LPS can subsequently be transported to other organs in the body, where its endotoxin activity can induce low-grade chronic inflammation linked to various metabolic diseases. In people with obesity, insulin resistance and type 2 diabetes, increased plasma levels of LPS were found compared to levels in healthy subjects after ingestion of a high-fat diet [15]. Moreover, LPS activity was reported to be strongly correlated with metabolic and cardiovascular risk factors and the number of components of the metabolic syndrome [16, 17]. This link between LPS and metabolic health risk is referred to as metabolic endotoxemia [18].

In addition to metabolic endotoxemia, there is growing evidence that (micro)-inflammation of the intestinal mucosa plays a role in the pathogenesis of irritable bowel syndrome (IBS) and inflammatory bowel disease (IBD) [19, 20]. IBS symptoms have been reported to correlate with increased permeability and subclinical inflammation [21]. Furthermore, Dlugosz and colleagues [22] have shown that patients with IBS-D (diarrhea predominant IBS subtype) have higher serum levels of LPS, indicative of subclinical inflammation [23, 24]. In IBD patients, elevated serum LPS was found to be associated with higher disease activity [19, 25]. Furthermore, an increased risk for developing IBD was linked to the intake of dietary fat [26], supporting a role for dietary fat-induced LPS translocation in IBD pathology.

To identify promising interventions that can prevent or ameliorate LPS-induced low-grade inflammation, in vitro models might be valuable for high-throughput screening. Previous studies have already indicated that intestinal epithelial Caco-2 cells can serve as an in vitro model to study fat-induced LPS translocation via chylomicron secretion [11, 27]. However, most of these studies used free fatty acids to induce chylomicron secretion and not the unrefined dietary oils that are typically part of the Western diet. The aim of our study was to develop an in vitro model for dietary fat-induced transcellular LPS translocation that more accurately mimics the in vivo situation. We investigated the effects of in vitro digested palm and sunflower oils on LPS translocation in Caco-2 cells.

## Methods

### Chemicals

All chemicals were purchased from Sigma Aldrich (St Louis, Missouri, MO, USA) unless otherwise stated.

### In vitro gastrointestinal digestion

First, palm oil (mainly C16:0) and sunflower oil (mainly C18:2) (Research Diet Services BV; for a more detailed composition, see Additional file 1) were both diluted to 0.2 g/mL with 140 mM NaCl + 5 mM KCl to a total volume of 15 mL. This mixture was gently vortexed and heated for 15 min at 56 °C. To mimic gastric digestion, the pH was set to 2 with 1 M HCl and 40 mg/mL pepsin solution (1092 U/mL dissolved in 0.1 M HCl) was added. The samples were incubated for 1 h at 37 °C while gently shaking. To mimic intestinal digestion, the pH was set to 5.8 with 1 M NaHCO_3_ and 4 mg/mL pancreatin (6.84 U/mg trypsin activity), 5.9 units/mL α-chromotrypsin (65.62 U/mg), 1 mg/mL lipase (all from porcine) and bile salts (94.6 mg/mL sodium taurocholate and 83 mg/mL sodium glycodeoxycholate) dissolved in 0.1 M NaHCO_3_ were added. Next, the pH was adjusted to 6.5 using 1 M NaHCO_3_, after which the samples were incubated at 37 °C for 2 h while gently shaking. After incubation, the pH was adjusted to 7.5 with 1 M NaHCO_3,_ and the volume of the digest was brought to 40 mL with 140 mM NaCl + 5 mM KCl. As a control, a digest was prepared that contained all buffers and enzymes but without the oils.

### Caco-2 culture

Caco-2 cells (ATCC-HTB-37) were cultured in Dulbecco’s modified Eagle’s medium (DMEM) with high glucose (4.5 g/L) and 25 mM HEPES (Life Technologies, 42,430) supplemented with 10% heat-inactivated fetal bovine serum (FBS, HyClone) at 37 °C and 5% CO_2_. A bicameral cell culture system with translucent, 0.4 μm cell culture inserts (Greiner Bio-one) were seeded on the apical side with 500 µL (12 wells) or 150 µL (24 wells) of 0.225 × 10^6^ cell/mL Caco-2, with 1500 µL (12 wells) or 750 µL (24 wells) of basolateral medium, respectively. The cells were incubated for 21 days at 37 °C with 5% CO_2_ to differentiate into small intestine-like epithelial cells. Apical and basolateral medium was replaced three times a week and one day prior to the investigational exposure. To monitor the integrity of the Caco-2 monolayer, transepithelial electrical resistance (TEER) was measured using a MilliCell ERS (Millipore Amsterdam, The Netherlands). TEER t = 0 is measured immediately after adding the tested compounds. The absolute TEER values of the control digest were highly stable over time. Differentiated Caco-2 monolayers were considered of acceptable quality if TEER values were higher than 700 Ω/cm^2^ (24 wells) and 450 Ω/cm^2^ (12 wells) before exposure to the investigational treatment.

### Fat-induced LPS translocation experiments

Prior to exposure to digested fats and LPS (Sigma L4391), Caco-2 cells were generally FBS deprived for one week, meaning that culture medium without added FBS was used in the apical compartment for the last 3 medium replacements. Subsequently, Caco-2 cells were apically exposed to digested oil samples (1:50 diluted in DMEM, unless stated otherwise) for 24 h. DMEM and control digest samples (also 1:50 diluted in DMEM, unless stated otherwise) were included as negative controls. To determine the fat-induced translocation of LPS, 100, 250, 500, 750, or 1000 ng/mL LPS was apically added for 24 h. All these exposure experiments were performed in DMEM without phenol red (Life Technologies) and FBS, as this can interfere with chylomicron detection. An MTT assay did not reveal any cytotoxic effect of the treatments (viability > 80%). The TEER was measured during the exposure experiments at 0, 1, 3, 6 and/or 24 h.

### Chylomicron blockage experiments

To investigate whether fat-induced LPS translocation is chylomicron-dependent in our model, we also performed Caco-2 experiments with and without the chylomicron blocker Pluronic L81 (PL81, BASF Corporation, Germany), as described by Ghoshal et al. [11].

Caco-2 cells were first preincubated overnight with LPS before exposure to PL81 and dietary fats to prevent paracellular LPS leakage by PL81 exposure. Therefore, 20-day differentiated Caco-2 cells deprived of apical FBS for 1 week were apically challenged overnight with 1 mg/mL LPS (diluted in DMEM without FBS and phenol red). Next, Caco-2 cells were washed 3 times with DMEM without FBS and phenol red, after which digested oil samples were apically added and mixed with 500 µg/mL PL81. After 24 h of incubation, chylomicron production and LPS translocation were assessed in the basolateral compartment.

### Detection of the chylomicron marker ApoB

Chylomicron production by Caco-2 cells was assessed by measuring apolipoprotein B (ApoB) levels in the basolateral medium using an ApoB ELISA (ABIN612664, Antibodies-online GmbH, Germany) according to the manufacturer’s protocol. This assay detects both ApoB-48 and ApoB-100. In in vivo settings, adult human intestines secrete chylomicrons that contain only ApoB-48, not ApoB-100, which is predominantly associated with VLDL derived from the liver [28]. However, Caco-2 cells are known to produce and secrete chylomicrons with both ApoB-48 and ApoB-100 [29, 30].

### Detection of LPS

LPS was measured in the apical as well in basolateral medium with a chromogenic Pyrochrome® assay (Associates Cap. Cod Inc, East Falmouth, MA, USA; distributed by Nodia BV, the Netherlands). The assay was performed in a microtiter plate reader (TECAN, Giessen, The Netherlands) as well as in a Pyros Kinetix Flex tube reader (pKFlex; Nodia BV, the Netherlands). Samples were diluted 25 times with LPS-free MQ and incubated for 15 min in a water bath at 70 °C. After 1 h at 4 °C, the samples were placed for 10 min at room temperature. Pyrochrome lysate was reconstituted with 3.2 mL Pyrochrome® reconstitution buffer (C1500-5; Associates Cap. Cod Inc, East Falmouth, MA, USA). After adding the Pyrochrome to the samples, the samples were directly measured with an endpoint measurement on the microtiter plate reader or on the pKFlex. For both methods, a calibration from Limulus Amebocyte lysate control standard endotoxin 0.5 µg/vial (CSE E0005-1; Associates Cap. Cod Inc, East Falmouth, MA, USA) was used. For the endpoint method, 125 µL Pyrochrome® was added to 125 µL treated sample or standard, and the mixture was incubated at 37 °C. After 35, 45 and 60 min, an 80 µL sample was taken and added to 20 µL acetic acid to stop the reaction. Finally, the absorbance was measured at 405 nm on a plate reader. For the pyros kinetix flex method, 200 µL of treated sample or standard was added to a pKflex glass tube. Fifty microliters of Pyrochrome® was added, and the mixture was briefly stirred and placed in pKFlex. Using the Pyros express 21 CFR Part 11 compliant software, the endotoxin concentration was calculated.

Another method used for functional LPS quantification is the HEK-Blue hTLR4 assay [31].

HEK-Blue hTLR4 cells are transgenic for the cell surface expressed hTLR4 MD-2 and CD14 receptors and contain a downstream reporter system resulting in secretion of secreted embryonic alkaline phosphatase (SEAP) under the control of NF-kB and AP1 promotors. HEK-Blue hTLR4 cells (InvivoGen, Toulouse, France) were cultured in DMEM with 10% FBS and subcultured once per week, and the medium was refreshed twice per week.

For experiments, HEK-Blue hTLR4 cells were detached using a cell scraper when 90–95% confluence was reached, and 0.5 × 10^6^ cells were transferred to each well of a 96-well poly-D-lysine-coated plate. After overnight incubation at 37 °C in an atmosphere containing 5% CO_2,_ we added 0.0001-100 EU/mL LPS (*E. coli* O11: B4, 1 EU = 0.15 ng/mL, Sigma, St. Louis, MO, USA) or 100 µL basolateral medium from the fat-induced LPS translocation experiments to the cells, and the plate was incubated for 8 h (37 °C; 5% CO_2_). TLR4 stimulation resulted in SEAP secretion, which was quantified by mixing 20 µL of supernatant (depleted from cells by centrifugation at 450 *g* for 5 min) with 180 µl of Quanti-Blue™ in a new flat bottom 96-well plate. The plate was incubated for 3 h at 37 °C, and absorption at 655 nm was determined every hour using a spectrophotometer (TECAN, Giessen, The Netherlands).

### Detection of paracellular translocation by FD4

To determine paracellular translocation of LPS in the Caco-2 exposure experiments, 250 µg/mL 4 kDa FITC-dextran (FD4) was added to the apical side at the start of the investigational exposures. After 24 h, fluorescence (excitation 485 nm/emission 528 nm) in the basolateral compartment was determined with a pleat reader (TECAN, Giessen, The Netherlands). The fluorescent values were compared to a calibration curve for FD4, and the percentage of paracellular translocation was calculated.

### Data analyses

Exposures performed with a concentration range to determine optimal conditions for the model were conducted as a single-replicate experiment. Verification exposures were performed in biological triplicate. These data are represented as the average ± SD. Statistical significance of differences was analyzed by ANOVA followed by Student’s *t* test for pairwise comparisons. Differences between groups were considered statistically significant when *P* < 0.05.

## Results

### Dietary fat-induced chylomicron secretion in Caco-2 cells

**Fig. 1:**
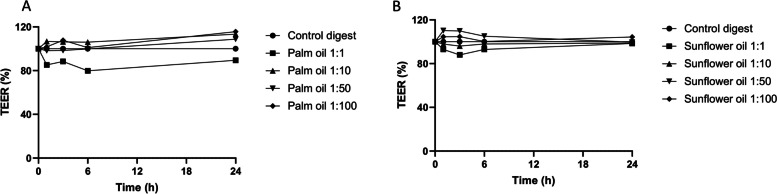
Effect of digested palm oil and sunflower oil dilutions on the intestinal integrity of Caco-2 cells. Effect of different dilutions of digested palm oil (**A**) and sunflower oil (**B**) on the intestinal integrity of Caco-2 cells, as assessed by TEER. TEER is expressed as % compared to the digest control (1:1), which was set to 100%. Digested oils and control digest were diluted in DMEM w/o phenol red and w/o FBS. Exposures were conducted as a single-cell experiment

**Fig. 2:**
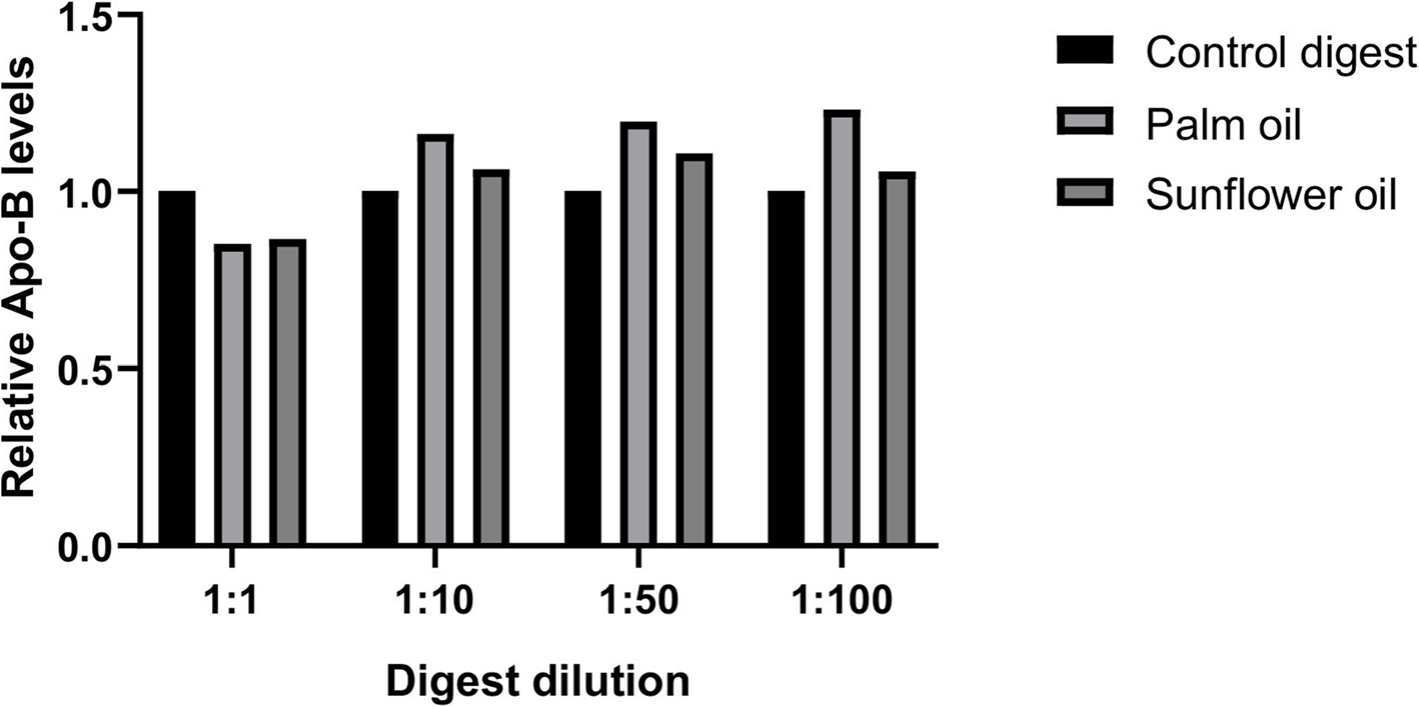
Effect of digested palm oil and sunflower oil dilutions on basolateral ApoB secretion by Caco-2 cells. Effect of different dilutions of control digest, digested palm oil and sunflower oil on basolateral ApoB secretion by Caco-2 cells. ApoB levels are expressed as relative levels compared to the control digest. Digested oils and control digest were diluted with DMEM w/o FBS and w/o phenol. Exposures were conducted as a single-cell experiment

To study the potential of saturated and unsaturated dietary fats to induce chylomicron production in Caco-2 cells, palm oil (mainly C16:0) and sunflower oil (mainly C18:2) were used, respectively. An in vitro digestion step was performed with these dietary fats before applying them to Caco-2 cells to better mimic the luminal conditions in the small intestine. The digested oils were tested at different dilutions to determine the optimal conditions for chylomicron production. The 1:10, 1:50, and 1:100 dilutions of digested oils did not appear to negatively impact the barrier integrity of small intestinal-like Caco-2 cells, as assessed by TEER. In contrast, the 1:1 dilutions reduced the TEER to some extent when compared to the control digest (Fig. 1 A and B), indicative of compromised intestinal integrity. This seems to fit with the production of chylomicrons, as only 1:10, 1:50 and 1:100 dilutions of digested palm oil and sunflower oil induced an increase in basolateral ApoB levels (Fig. 2). At all these concentrations, palm oil showed the most pronounced induction of ApoB, namely, an approximate 1.2-fold increase (20%) compared to the digest control. The (middle) 1:50 dilution was selected to continue optimization of the model, as Luchoomun et al. previously showed that too high or too low levels of fatty acids (in combination with bile acids) can result in reduced ApoB secretion by Caco-2 cells [27].

**Fig. 3:**
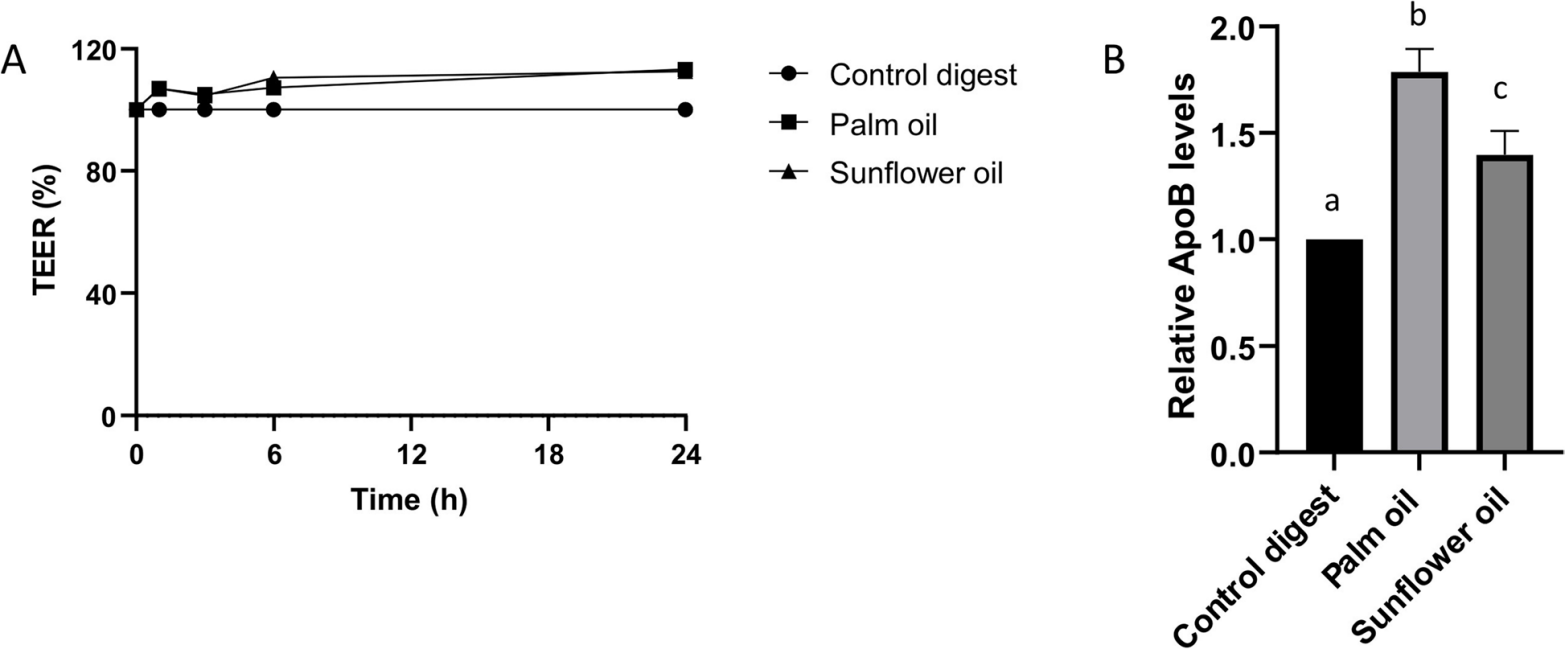
Effect of 1 week of apical FBS deprivation prior to digested palm oil and sunflower oil exposure on the intestinal integrity and basolateral ApoB secretion of Caco-2 cells. **A** Effect of digested palm oil and sunflower oil (both 1:50 diluted) on the intestinal integrity of Caco-2 cells, as assessed by TEER, preceded by 1 week of apical FBS deprivation. TEER is expressed as % compared to the control digest (also 1:50 diluted), which was set to 100%. Digested oils and control digest were diluted with DMEM w/o FBS and w/o phenol. Exposures were conducted as 3 biological replicates (*N* = 3). **B** Effect of digested palm oil and sunflower oil (both 1:50 diluted) on basolateral ApoB secretion by Caco-2 cells after 1 week of apical FBS deprivation. ApoB levels are expressed as relative levels compared to the control digest (also 1:50 diluted). Exposures were conducted in biological triplicate (*N* = 3); a, b, c represent significant differences between the treatments (*P* < 0.05)

To mimic the in vivo situation more closely [32], Caco-2 cells were FBS deprived (meaning deprived from FBS on the apical side) in the last week of differentiation and thus one week before dietary fat exposure. After 24 h of exposure to the 1:50 diluted digested oils, the intestinal integrity of the apical FBS-deprived Caco-2 cells was not affected when compared to the control digest (Fig. 3A). In contrast, chylomicron secretion was significantly increased 1.7 fold by palm oil compared to the digest control (Fig. 3B). This indicates that apical FBS deprivation of Caco-2 cells prior to fat exposure increases chylomicron production, as a higher induction was now seen (1.7 fold induction versus 1.2 fold without FBS deprivation, Fig. 2), thereby enlarging the window of opportunity for modulation. Based on these data, we concluded that a 1:50 dilution of in vitro digested oils and 1-week apical FBS deprivation of the Caco-2 cells prior to fat exposure were the best conditions for fat-induced chylomicron secretion in our Caco-2 cell model.

### Dietary fat-induced LPS translocation in Caco-2 cells

**Fig. 4:**
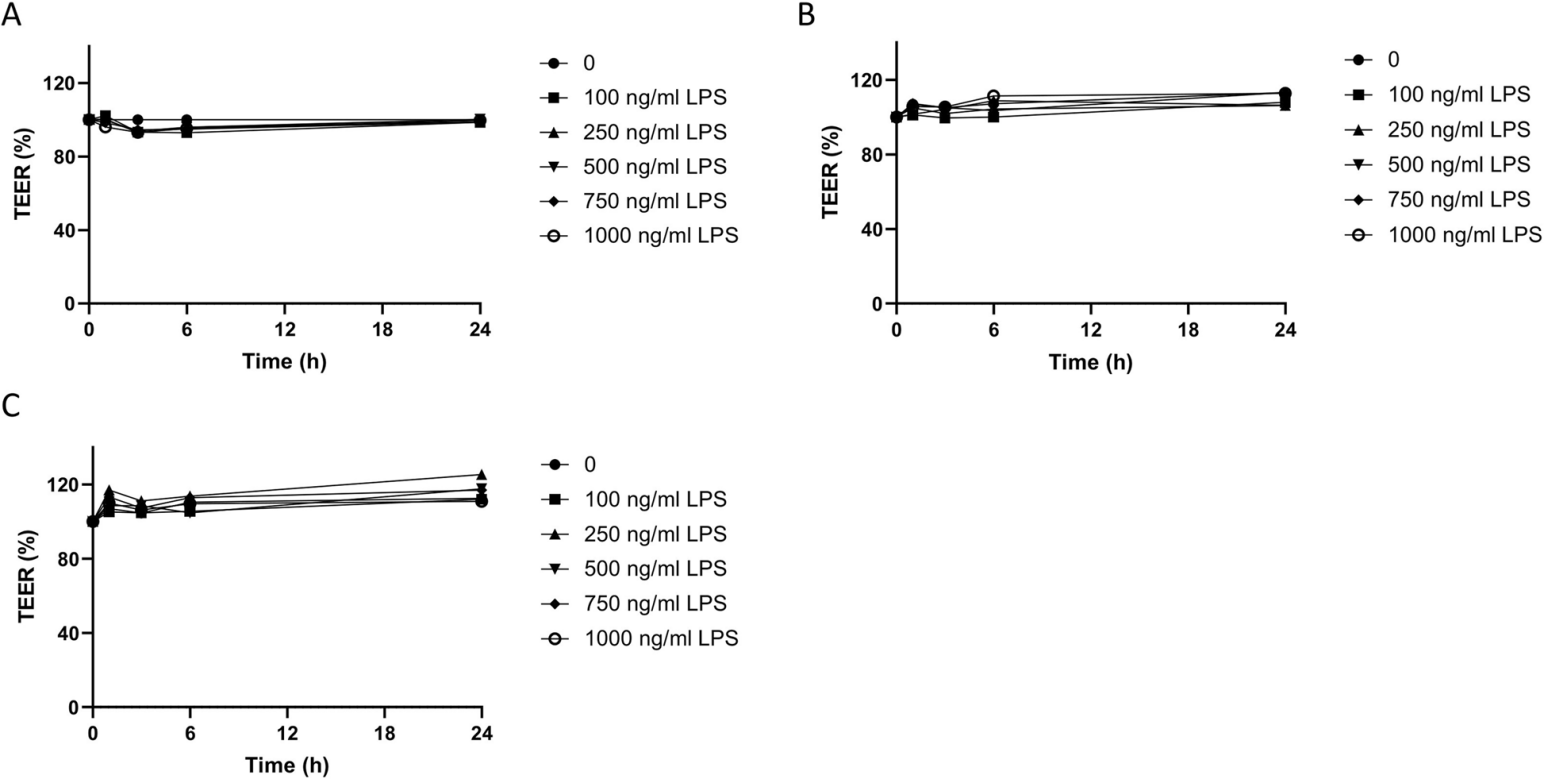
Effect of LPS concentrations on the intestinal integrity of Caco-2 cells coexposed to digested palm oil and sunflower oil. Effect of different concentrations of LPS (ng/mL) on intestinal integrity (TEER) of Caco-2 cells exposed to digested fats and 1 week apical FBS deprivation TEER is expressed as % compared to digest control (100%). Digest control (**A**), digested palm oil (**B**) and digested sunflower oil (**C**) were diluted 1:50 in DMEM w/o phenol red and w/o FBS. Caco-2 cells were coincubated after 1 week of apical FBS deprivation with 0, 100, 250, 500, 750, and 1000 ng/mL LPS. These exposures were conducted as a single-cell experiment

**Fig. 5:**
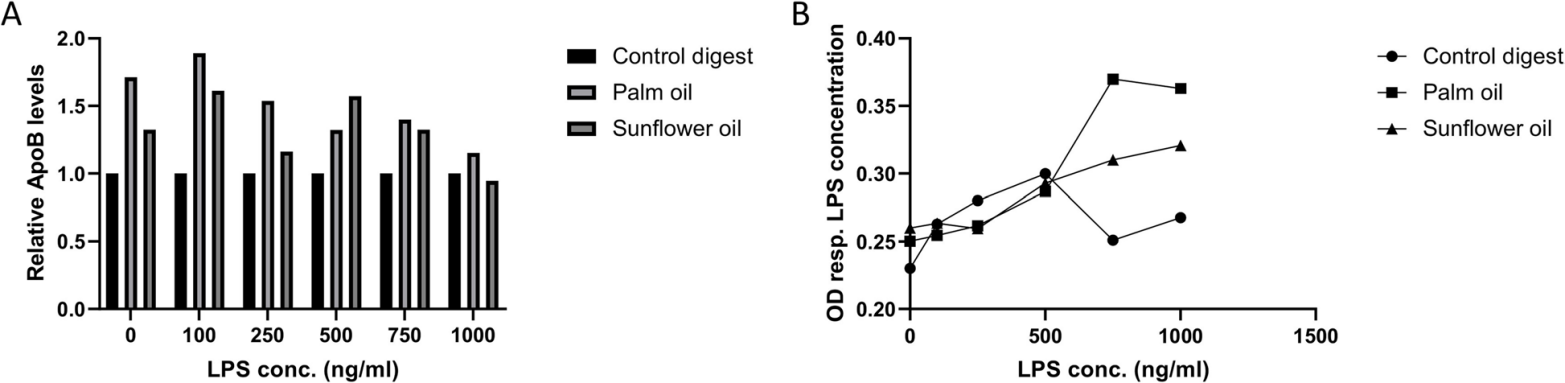
Effect of LPS concentrations on basolateral ApoB secretion and LPS translocation by Caco-2 cells after coexposure to digested palm oil and sunflower oil. Effect of different concentrations of LPS (ng/mL) on ApoB secretion (**A**) and LPS translocation (**B**) by fat-exposed Caco-2 cells. Digest control, digested palm oil and digested sunflower oil were diluted 1:50 in DMEM w/o phenol red and w/o FBS. Caco-2 cells were coincubated with LPS concentrations of 0, 100, 250, 500, 750, and 1000 ng/mL for 24 h. LPS was measured with the HEK-TLR4 assay. The OD represents the LPS concentration in the basolateral samples. Exposures were conducted as a single-cell experiment

Having established the optimal culture and fat exposure conditions to stimulate fat-induced chylomicron secretion in Caco-2 cells, we investigated whether this model could be extended to study dietary fat-induced LPS translocation. To this end, Caco-2 cells were coincubated with the digested oils and different concentrations of LPS (0, 50, 100, 250, 500, 750 or 1000 ng/mL). A 24 h exposure to these samples did not affect intestinal integrity, as assessed by TEER, indicating that apical LPS has no detrimental effect on Caco-2 cells (Fig. 4). Furthermore, LPS does not seem to drastically affect fat-induced ApoB secretion, although there seems to be a slight reduction upon higher LPS concentrations (Fig. 5A). For LPS translocation, only at a high level of apical LPS exposure (≥ 750 ng/mL) was a clear distinction in basolateral LPS found between the digested oil samples and the control digest (Fig. 5B). This indicates that at least 750 ng/mL LPS is needed in combination with digested oils to induce dietary fat-induced LPS translocation in our Caco-2 cell model. Therefore, we continued the experiments using 750 ng/mL LPS.

**Fig. 6:**
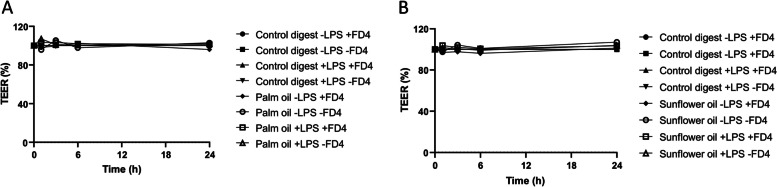
Effect of digested palm oil, digested sunflower oil, FD4 and LPS on the intestinal integrity of Caco-2 cells. Effect of digested palm oil (**A**) and sunflower oil (**B**), FD4 and LPS on intestinal integrity (TEER) of Caco-2 cells. TEER is expressed as % compared to the control digest without FD4 and LPS, which was set to 100%. Exposures were conducted as a single-cell experiment

**Fig. 7:**
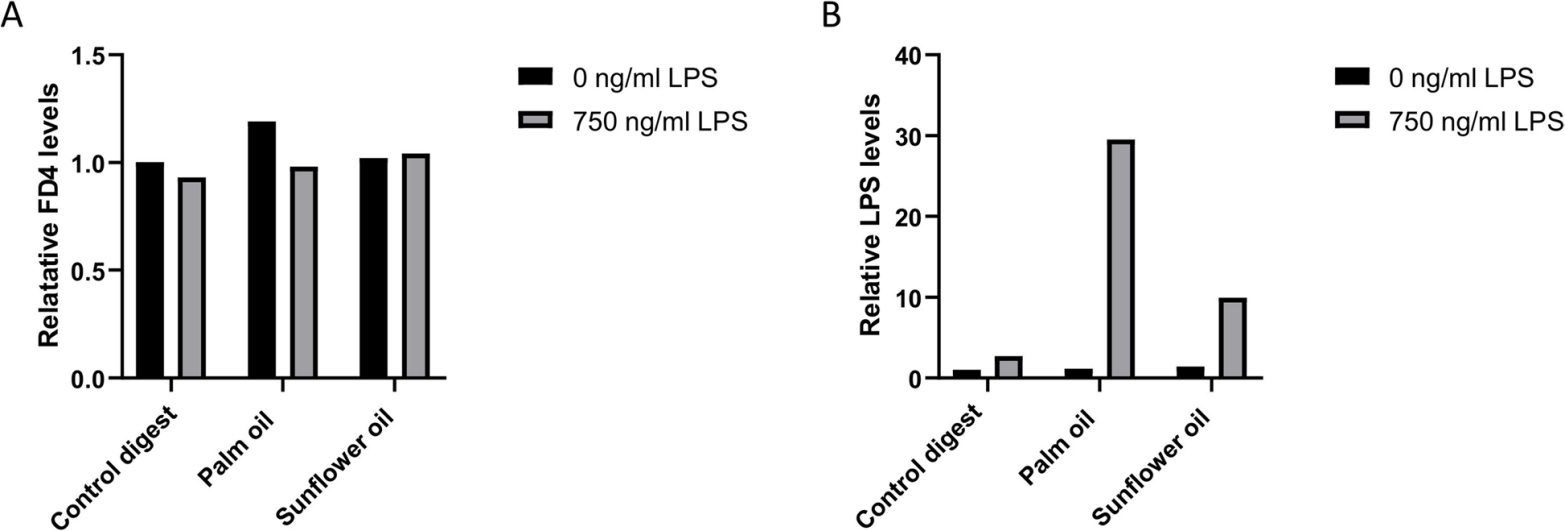
Effect of digested palm oil and sunflower oil on paracellular and transcellular translocation by Caco-2 cells. Effect of digested palm oil and sun flower oil on translocation of FD4 (**A**) and LPS (**B**) to the basolateral side of Caco-2 cells. Digest control, digested palm oil and digested sunflower oil were diluted 1:50 in DMEM w/o phenol red and w/o FBS. Caco-2 cells were coincubated with digested oils (1:50), 0 and 750 ng/mL LPS and 250 µg/ml FD4 for 24 h. Basolateral FD4 was calculated using a calibration curve and compared to the control digest. LPS was measured using the Pyrochrome® assay. These exposures were conducted as a single-cell experiment

To exclude paracellular LPS translocation, FD4 was apically added to the digested oils (1:50 dilution) and LPS (750 ng/mL) in the Caco-2 cell model. Again, no effect was observed on TEER and thus intestinal integrity (Fig. 6). After 24 h of exposure, no FD4 translocation to the basolateral compartment was detected, whereas basolateral LPS levels were still increased by palm oil and sunflower oil (Fig. 7 A and B). This indicates that the paracellular route is not involved in fat-induced LPS translocation in this Caco-2 cell model.

### Dietary fat-induced LPS translocation is mediated by chylomicron production in Caco-2 cells

**Fig. 8:**
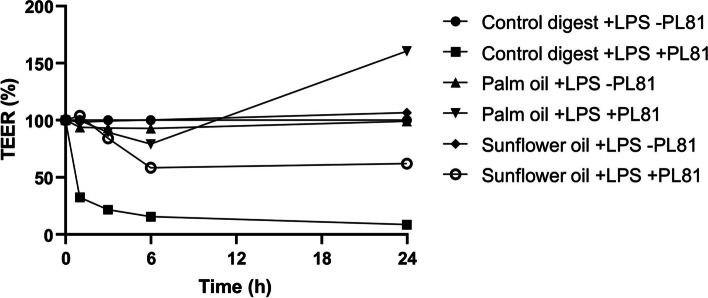
Chylomicron blockage by PL81 and its effect on the intestinal integrity of Caco-2 cells. Effect of PL-81 with or without palm oil and sunflower oil on intestinal integrity (TEER) in digested fat (1:50) and LPS-exposed Caco-2 cells. Caco-2 cells were incubated overnight with LPS (1 mg/mL). TEER t = 0 is measured immediately after adding oils and PL-81. TEER is expressed as % compared to the control digest with LPS and without PL-81, which was set to 100%. These exposures were conducted as 3 biological replicate (*N* = 3) experiments

To verify that dietary fat-induced LPS translocation is mediated by chylomicron production, the chylomicron blocker PL81 was added to the Caco-2 cell model. As PL81 is known to affect intestinal barrier function [33], the timing of exposures in the model was adapted to avoid paracellular leakage of LPS due to PL81-induced barrier disruption. Cario et al. described that LPS can be internalized and stored in intestinal epithelial cells [34]; therefore, Caco-2 cells were preincubated overnight with LPS instead of directly coincubated with digested dietary fats. Prior to coexposure to PL81, we first confirmed that LPS stored overnight could still be released upon fat exposure in Caco-2 cells. Coexposure of the Caco-2 cells with PL81 showed an expected strong reduction in TEER (Fig. 8), especially in control digest conditions. Surprisingly, a less dramatic drop in TEER was observed in cells coincubated with the oils. In particular, palm oil seemed to protect against the PL81-induced deterioration of the intestinal layer.

**Fig. 9:**
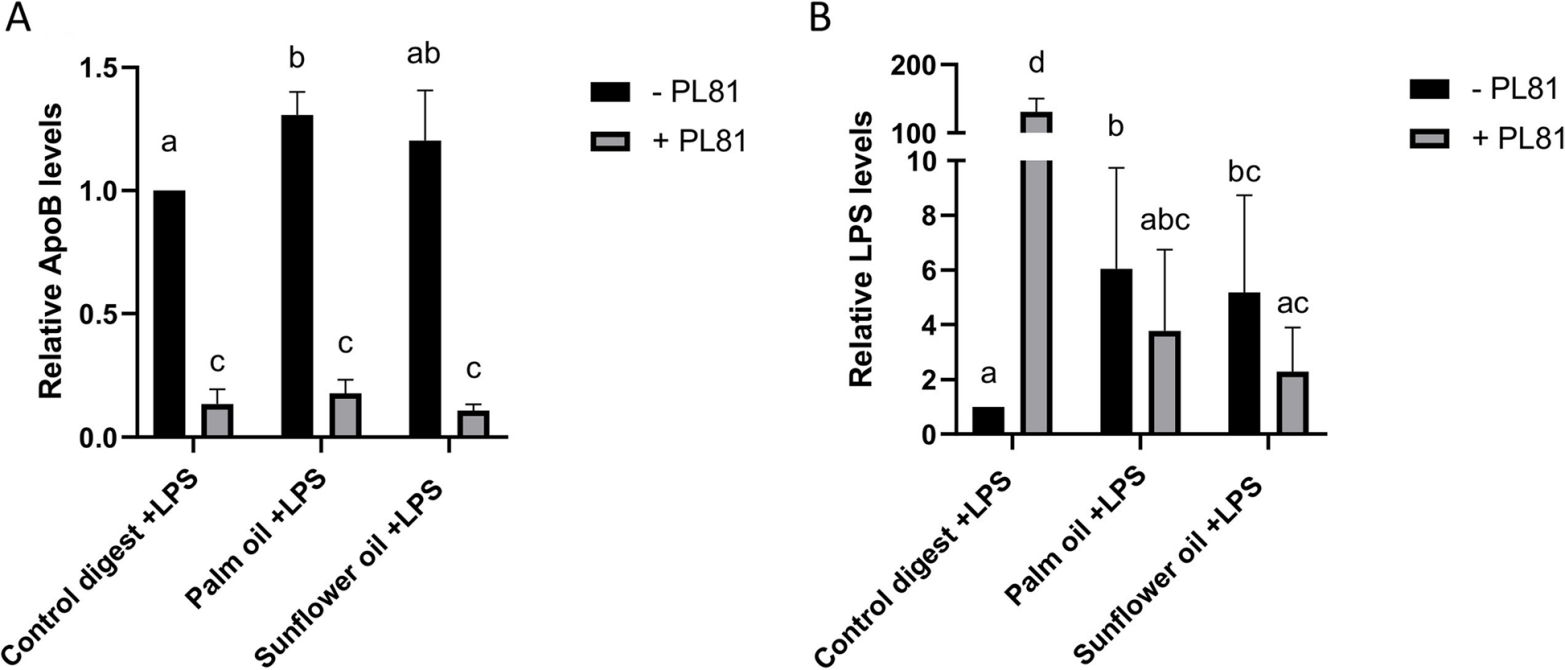
Chylomicron blockage by PL81 and its effect on basolateral ApoB secretion and LPS translocation. Effect of PL81 with or without palm oil and sunflower oil on Apo-B secretion (**A**) and LPS translocation (**B**) by Caco-2 cells after 1 week of apical FBS deprivation. Caco-2 cells were preincubated with 0 and 1000 ng/mL LPS overnight. Digest control, digested palm oil and digested sunflower oil were diluted 1:50 in DMEM w/o phenol red and w/o FBS with 500 µg/mL PL81. LPS was measured using the Pyrochrome® assay. These exposures were conducted as 3 biological replicates (*N* = 3) (*P* < 0.05)

Next, we measured the effect of PL81 on basolateral ApoB secretion (Fig. 9A) and LPS translocation (Fig. 9B) and found that PL81 significantly reduced basolateral ApoB levels, independent of the different digest samples. This indicates that PL81 accurately blocked chylomicron production in our Caco-2 cell model. When measuring LPS translocation upon PL81 coexposure, we observed a strong increase in basolateral LPS when Caco-2 cells were exposed to the digest control (Fig. 9B). This is probably linked to the strong disruption of the barrier integrity (Fig. 8) and thus mainly represents paracellular leakage. Interestingly, when Caco-2 cells were coexposed to either palm oil or sunflower oil digests, PL81 treatment (Fig. 9B; gray bars) even reduced LPS translocation in Caco-2 cells compared to that without PL81 treatment (Fig. 9B; black bars). Although this reduction in basolateral LPS did not reach significance (*P* = 0.1), the data suggest that dietary fat-induced LPS translocation in our model is (at least partly) mediated by chylomicron production.

## Discussion

We developed an in vitro Caco-2 model to study digested dietary fat-induced LPS translocation, which can serve as a screening tool to identify (food) compounds with potential beneficial and preventive effects on LPS-related local and systemic low-grade inflammation.

In this study, we determined the optimal concentrations of digested oils and LPS for apical exposure of Caco-2 cells to stimulate chylomicron production and subsequent LPS translocation without inducing cytotoxic effects and disrupting the intestinal barrier. Optimization experiments were only performed in a single experiment, which might be a statistical limitation. However, the optimal conditions chosen based on these single experiments were all validated in at least a biological triplicate to confirm their relevance. As both TEER and FD4 were not affected after exposure to digested oils and LPS, the transport of LPS is unlikely to be facilitated through paracellular transport. Similarly, Mani et al. [35] observed no difference in intestinal integrity in pigs after ex vivo treatments with endotoxins in relation to dietary oil compositions. Taken together, these data suggest that fat-induced LPS translocation is routed via transcellular transport and not paracellular transport.

Hiebl et al. [32] previously implied that asymmetric culturing conditions during the differentiation of Caco-2 cells more closely mimic the in vivo situation. They cultured the Caco-2 cells without FBS in the apical compartment of the bicameral system for the last part of the 21-day differentiation period. In the in vivo situation, the intestinal lumen is normally also not exposed to serum or serum-related compounds, so we chose to investigate the effect of asymmetric culture conditions in our Caco-2 cell model. We found that apical FBS deprivation in the last week before dietary fat exposure enhanced fat-induced chylomicron production, thereby enlarging the window of opportunity for modulation. Therefore, we consider this asymmetric culture condition an important element of our model.

The use of PL81, a known inhibitor of chylomicron production [11], suggested that chylomicron production is involved in fat-induced LPS translocation, as the dietary fat-induced secretion of ApoB was diminished with a consequent reduction in LPS translocation. PL81 uptake by differentiated Caco-2 cells is known to be a protein-facilitated active process, and PL81 inhibits fat absorption by decreasing triacylglycerol transport from the cytosol to the endoplasmic reticulum. This in turn inhibits the assembly and secretion of chylomicrons [33]. In the case of adding PL81 without dietary fat (digest control), we found that the integrity of the Caco-2 monolayer was severely disturbed as the TEER was considerably decreased. In other studies that use PL81 as a chylomicron inhibitor, this barrier disruption was not mentioned. However, our study shows that this is important to take into account when using this chylomicron blocking compound, especially when it is not used in combination with lipid-like molecules in the apical compartment. Upon coincubation with digested oils, we found that the disruption of the intestinal barrier by PL81 was much less pronounced, indicating a ‘protective effect’ of the digested fats against PL81-induced barrier disruption. The mechanism behind this is unclear, but it might be related to the cellular distribution of PL81 that can be affected by coincubation with dietary fat (ty acids) [33].

Fatty acid-induced chylomicron production and LPS translocation were previously studied in Caco-2 cells using free fatty acids by Luchoomun [27] and Ghoshal et al. [11], respectively. Incubation with in vitro digested oils more closely mimics the in vivo situation and should therefore be preferred over the fatty acid-induced LPS translocation model. Furthermore, it was previously reported that exposure of Caco-2 cells to palmitic acids alone can disrupt the intestinal barrier and thereby reduce ApoB secretion [36] and induce paracellular transport [37], which is not desirable for a fat-induced LPS translocation model. Luchoomun et al. [27] previously showed that bile acids are also important for efficient chylomicron production in Caco-2 cells. In our in vitro fat digestion protocol, the addition of bile acids is also included. Therefore, as part of the total digest, bile acids are also coexposed to Caco-2 cells in our cell model. Ghoshal et al. [11] showed that LPS translocation is dependent on long-chain fatty acid exposure in Caco-2 cells, whereas short-chain fatty acids that are not transported via chylomicrons [38] did not induce LPS transport. This indicates that chylomicron production is involved in LPS translocation, which was supported by their PL81 experiments abolishing the long-chain fatty acid-induced effect. Similar effects of PL81 were also found in our Caco-2 cell model, indicating that with digested oils, fat-induced LPS translocation is linked to chylomicron production. However, our data suggest that this LPS translocation induced by digested oils is only partly dependent on chylomicron production, as ApoB secretion was almost completely abolished by PL81, whereas LPS was only reduced by approximately 50%. This would, however, fit with recent findings of Akiba et al. [39], who showed a second lipid-dependent route for LPS transport in the intestine, which is independent of chylomicron secretion [39]. In their rat model, they also found that PL81 could not completely block fat-induced LPS translocation, and they additionally found (labeled) LPS back in the portal vein. They found no evidence for paracellular leakage of LPS. Alternatively, it has previously been shown that high fat consumption can induce internalization of tight junction proteins, which increases paracellular transport, including LPS transport [35]. This potential process for fat-induced LPS translocation was not reflected in TEER levels and FD4 translocation in our model. Based on our findings, however, we cannot exclude that paracellular leakage of LPS might still play a role in fat-induced LPS translocation.

Several in vivo studies have shown that the composition of dietary oil has a substantial effect on postprandial endotoxemia. In particular, saturated fats are linked to higher postprandial LPS levels in blood [35, 40–42]. This is in line with the results that we found in our model, as we also showed that, even though the difference between the fat types was sometimes subtle, palm oil more strongly induced chylomicron secretion and LPS translocation when compared to sunflower oil. In contrast, Laugerette et al. [12] showed that palm oil had no effect on plasma endotoxin concentrations, while sunflower oil augmented plasma endotoxemia by 59–70%. However, despite these findings, the authors concluded that palm oil has a more proinflammatory effect than unsaturated fat, based on higher plasma levels of other inflammatory markers. This implies that the conditions that were chosen in our model are accurate for studying fat-induced LPS translocation, although direct extrapolation to the in vivo situation is difficult, as is the case for most in vitro cell models. For future research, it would also be interesting to explore the potential differences in fat-induced LPS translocation between dietary fats high in n-6 and n-3 unsaturated fatty acids, as these are known to be more pro- and anti-inflammatory, respectively.

In Western diets, fats and oils are common components of the diet [43]. In recent years, the development of obesity, inflammation and other metabolic diseases has been linked to low-grade endotoxemia associated with high dietary fat and energy intake [16]. These studies have raised questions whether diet-induced endotoxemia is caused by changes in the permeability of the intestine, variations in the gut microbiota or simply by a change in the intake of the dietary fat and energy content of the food. There is increasing evidence that low-grade endotoxemia is induced by a combination of all the above factors. For instance, in addition to the effect of dietary fat on LPS translocation, a high saturated fat diet, such as palm oil, is reported to result in higher gram-negative bacterial populations, such as *E. coli*, which produce gut LPS [44]. Lifestyle changes, especially dietary interventions that reduce chylomicron production and lower LPS production by the gut microbiome, are thought to be effective in reducing low-grade inflammation and thereby reducing the risk for CVD and other comorbidities linked to metabolic syndrome.

In addition to this impact on metabolic health, gastrointestinal health has a link with dietary fat and LPS-related inflammation. Associations between fatty meal consumption and IBS symptoms have also been identified in a number of studies. In fact, in many IBS patients, the symptoms were triggered after the consumption of greasy or fried food [45, 46]. Dietary fat is also linked to an increased risk for developing IBD [26]. Interestingly, higher serum and/or fecal LPS levels are also found in IBD and IBS patients [47], especially in patients with predominant diarrhea-related symptoms [22]. This link with high fat intake and the previously found association with high LPS levels might indicate a role for fat-induced LPS translocation in IBS and IBD pathology. If confirmed in future studies, dietary interventions that influence fat-induced LPS translocation might also be beneficial for this patient population.

## Study strengths and limitations

### Strengths

- Our study demonstrates the reprodicibility of fat-induced LPS translocation in an accesible in vitro Caco-2 cell model,
- Results of our Caco-2 cell model are highly comparable to in vivo data in literature, indicating that the use of the digestion and exposure methods in combination with the tested oil samples show relevance to real-life applications.
- The use of PL-81 provides mechanistic insights into fat-induced LPS translocation in relation to chylomicron production.
- The Caco-2 cell model and materials are widely available, making it a suitable model for medium/high-throughput screening.

### Limitations

- The use of single replicates for optimization of the model can be considered a limitation of the study. However, validation of the model is always performed in triplicate, ensuring reproducibility of the results.
- Inability for simultaneous exposure of LPS and PL-81 in our Caco-2 cell model possibly underestimates the importance of chylomicron production in fat-induced LPS translocation. However, our results are still in line with in vivo data in literature.
- Fat-induced LPS translocation is now only studied in Caco-2 cells, without testing other intestinal epithelial cell lines or primary human cells. Also the use of organoids could be an interesting option to further explore in future research.
- For now, the Caco-2 cell model is only tested with oils, so no conclusions can be drawn on the use of full food matrices.

## Conclusion

We developed an in vitro model that closely mimics the in vivo situation to study dietary fat-induced LPS translocation linked to LPS-related local or systemic low-grade inflammation. This Caco-2 cell model can be used to screen dietary fats and other food components that can prevent or reduce inflammatory conditions. In this way, this model contributes to research identifying beneficial food (compounds) for human metabolic and gastrointestinal health.

## Supplementary Information

**Additional file 1.**

## Data Availability

All data generated or analyzed during this study are included in this published article.
