# Supplementary material for: Dietary fat induced chylomicron-mediated LPS translocation in a bicameral Caco-2cell model
Source: Lipids Health Dis. 2023 Jan 12;22:4. doi: 10.1186/s12944-022-01754-3 (PMC9835336; doi:10.1186/s12944-022-01754-3)
Supplement: Supplementary file 1 — Additional file 1. [file 12944_2022_1754_MOESM1_ESM.docx]

| Fatty acids | Amount (%) | |
| --- | --- | --- |
|  | **Palm oil** | **Sunflower oil** |
| 12:0 (Lauric acid) | 0.4 ± 0.07 | 0.7 ± 0.00 |
| 14:0 (Myristic acid) | 1.1 ± 0.04 | 0.87 ± 0.00 |
| 16:0 (Palmitic acid) | 42.14 ± 0.13 | 5.29 ± 0.02 |
| 18:0 (Stearic acid) | 4.4 ± 0.01 | 4.75 ± 0.05 |
| 18:1 (Oleic acid) | 38.23 ± 0.2 | 11.37 ± 0.08 |
| 18:2 (Linoleic acid) | 10.78 ± 0.02 | 73.24 ± 0.35 |
| 18:3 (Linolenic acid) | – | – |
| Other Minors | 2.95 ± 0.00 | 3.78 ± 0.04 |
| SFA | 50.45 | 11.61 |
| USFA | 49.55 | 88.39 |

**Additional file 1. Fatty acid composition of palm oil and sunflower oil.**

*SFA: saturated fatty acids, USFA: unsaturated fatty acids*

*Based on: Devi et al. J Food Sci Technol. 2018 Jan; 55(1): 321–330*
